# Potential impact and cost-effectiveness of *Shigella* vaccination in 102 low-income and middle-income countries in children aged 5 years or younger: a modelling study

**DOI:** 10.1016/S2214-109X(23)00192-4

**Published:** 2023-05-16

**Authors:** John D Anderson, Karoun H Bagamian, Clint J Pecenka, Farzana Muhib, Chloe A Puett, William P Hausdorff, Suzanne Scheele

**Affiliations:** a Bagamian Scientific Consulting, Gainesville, FL, USA; b Health Affairs Institute, West Virginia University, Morgantown, WV, USA; c Department of Environmental and Global Health, University of Florida, Gainesville, FL, USA; d Center for Vaccine Innovation and Access, PATH, Seattle, WA, USA; e Center for Vaccine Innovation and Access, PATH, Washington, DC, USA; f Stony Brook University, Department of Family, Population & Preventative Medicine, Program in Public Health, Stony Brook, NY, USA; g Faculty of Medicine, Université Libre de Bruxelles, Brussels, Belgium

## Abstract

**Background:**

Vaccine impact and cost-effectiveness models have mostly focused on acute burden. *Shigella*-attributable moderate-to-severe diarrhoea has been shown to be associated with childhood linear growth faltering. Evidence also links less severe diarrhoea to linear growth faltering. As *Shigella* vaccines are in late stages of clinical development, we aimed to estimate the potential impact and cost-effectiveness of vaccination against *Shigella* burden that includes stunting and the acute burden attributable to less severe diarrhoea and moderate-to-severe diarrhoea.

**Methods:**

We used a simulation model to estimate *Shigella* burden and potential vaccination in children aged 5 years or younger from 102 low-income to middle-income countries from 2025 to 2044. Our model included stunting associated with *Shigella*-related moderate-to-severe diarrhoea and less severe diarrhoea and we explored vaccination impact on health and economic outcomes.

**Findings:**

We estimate 109 million (95% uncertainty interval [UI] 39−204) *Shigella*-attributable stunting cases and 1·4 million (0·8–2·1) deaths in unvaccinated children over 20 years. We project that *Shigella* vaccination could avert 43 million (13−92) stunting cases and 590 000 (297 000–983 000) deaths over 20 years. The overall mean incremental cost-effectiveness ratio (ICER) was US$849 (95% uncertainty interval 423–1575; median $790 [IQR 635–1005]) per disability-adjusted life-year averted. Vaccination was most cost-effective in the WHO African region and in low-income countries. Including the burden of *Shigella*-related less severe diarrhoea improved mean ICERs by 47−48% for these groups and substantially improved ICERs for other regions.

**Interpretation:**

Our model suggests that *Shigella* vaccination would be a cost-effective intervention, with a substantial impact in specific countries and regions. Other regions could potentially benefit upon the inclusion of the burden of *Shigella*-related stunting and less severe diarrhoea in the analysis.

**Funding:**

Bill & Melinda Gates Foundation and Wellcome Trust.

## Introduction

Traditional disease burden measures, vaccine impact analyses, and clinical trials generally focus on acute burden (primarily mortality). However, for enteric infections, downstream health effects of an acute episode can substantially affect an individual's health, quality of life, and survival. One of the potentially most detrimental long-term sequelae of enteric infections is linear growth faltering, when a child's height falls below the expected growth curve. Stunting is a specific case of linear growth faltering, when a child's height-for-age Z score (HAZ) is 2 SDs below WHO's child growth standards. Linear growth faltering and stunting are associated with important short-term and long-term outcomes in a child's life, such as increased susceptibility to other infections, exacerbation of undernutrition, physical and cognitive development delays, and reduced educational attainment and economic productivity.^1^, ^2^, ^3^ Researchers have subsequently expanded the burden definition for enteric diseases to include indirect and long-term effects; these expanded analyses report substantial burden and medical cost increases, indicating need for a broader approach.^4^, ^5^, ^6^, ^7^

Before the availability of more sensitive molecular diagnostics, empirical studies primarily focused on moderate-to-severe diarrhoeal disease when evaluating long-term impacts,^8^ with burden and cost-effectiveness models taking the same approach.^6^, ^7^ However, the increased use of molecular diagnostics in large-scale epidemiological studies has shown that less severe enteric illness is also associated with childhood linear growth faltering,^9^, ^10^ even showing that subclinical infections affect child growth.^10^ *Shigella* specifically has been found to have a robust relationship to childhood linear growth faltering.^10^, ^11^, ^12^, ^13^ *Shigella* is also associated with markers of environmental enteric dysfunction^12^, ^14^—the proposed link between enteric infection and growth deficits. Additionally, *Shigella* is the second leading cause of all-cause diarrhoeal mortality among all age groups (13% of all diarrhoeal deaths), accounting for almost 64 000 deaths among children aged 5 years or younger worldwide annually;^15^ *Shigella* has the highest incidence in young children after infancy (age 12–59 months),^8^, ^9^ and *Shigella*-attributable diarrhoea can have especially severe symptoms (eg, dysentery and protein-losing enteropathy).

**Research in context**

**Evidence before this study**

We searched PubMed from inception to June 29, 2022, without language or location restrictions, using two search strings: one for the diarrhoeal disease aetiology in children younger than 5 years and another on diarrhoea and stunting. For diarrhoeal aetiology in children younger than 5 years, we found 74 publications with the search string (“shigella” OR “shigellosis”) AND (“children under five” OR “children under 5” OR “children <5”) AND (“morbidity” OR “illness” OR “stunting” OR “episodes” OR “events” OR “cases”) AND (“cause” OR “etiology” OR “aetiology”). For diarrhoea and stunting, we found 271 publications with the search string (“diarrhea” OR “diarrea” OR “diarrhoea”) AND (“stunting” OR “height for age” OR “height” OR “Z score” OR “linear growth faltering”) AND (“child*” OR “children under five” OR “children under 5” OR “children <5”) AND (“outcomes” OR “changes”). Two large-scale multisite studies—the Global Enteric Multicenter Study (GEMS) and the Malnutrition and Enteric Disease Study (MAL-ED)—have found that less severe *Shigella*-associated disease can also affect child growth. While several *Shigella* vaccines are in development, four studies have explored *Shigella* burden or cost-effectiveness of *Shigella* vaccination in children younger than 5 years. However, in our literature search, we did not find any studies that estimated the burden of less severe *Shigella*-attributable diarrhoea and stunting or associated vaccine impact and cost-effectiveness for children from low-income and middle-income countries (or any other population).

**Added value of this study**

We estimated *Shigella*-attributable morbidity, mortality, and stunting from episodes of less severe and moderate-to-severe diarrhoea for children younger than 5 years living in 102 low-income and middle-income countries. We adapted and refined our previously published approach with methods for including the diarrhoeal and stunting burden of less severe *Shigella*-attributable disease and estimated the health and economic impacts of reducing rates of less severe and moderate-to-severe *Shigella* disease through vaccination. Our analysis showed that *Shigella* vaccines are projected to be cost-effective in certain high-burden areas, even when using the most conservative model that only included moderate-to-severe episodes. Upon the inclusion of less severe episodes and the stunting-associated burden, vaccination was cost-effective in even more areas.

**Implications of all the available evidence**

Our study further illustrates the importance of including the broader effects of stunting for potential *Shigella* (and other enteric) vaccines when assessing their value. Having a broader approach allows countries or policy makers to have a more comprehensive picture of vaccination benefits—providing a best-case scenario—which can help guide policy decisions.

*Shigella* is a WHO priority vaccine target because of its high burden and its high antibiotic resistance.^16^ Although no *Shigella* vaccines are approved for use, many are in the late stages of development.^16^ The potential for a *Shigella* vaccine to improve acute and long-term health in young children from high-risk settings and mitigate further antibiotic resistance highlights the importance of characterising the broader *Shigella*-related burden and vaccine impact.

Here, we explored how including episodes of less severe *Shigella* diarrhoea and their impact on childhood stunting and related mortality influence *Shigella* burden and vaccination-related effects. We adapted and expanded a previous model^6^, ^7^ to include the additional childhood burden from less severe *Shigella* infections, using updated methods and estimates. Then, we estimated the health and economic impacts over 20 years of potential *Shigella* vaccination, comparing the overall influence of moderate-to-severe and less severe diarrhoea on health outcomes, medical costs, and vaccine cost-effectiveness.

## Methods

### Overview

In this modelling study, we adapted and expanded our previous *Shigella* burden and impact models^6^, ^7^ to explore health and economic effects of future *Shigella* vaccination in children aged 5 years or younger from 102 low-income, lower-middle-income, and upper-middle-income countries (appendix p 5). First, we assessed *Shigella*-attributable morbidity (episodes of less severe diarrhoea and moderate-to-severe diarrhoea and *Shigella*-attributable childhood stunting) and mortality burden (total *Shigella* deaths, which included deaths from acute *Shigella* episodes and from other infections due to *Shigella*-attributable stunting).^6^ We estimated health (averted mortality, episodes, and stunting) and economic effects (medical costs and vaccine cost-effectiveness) of *Shigella* vaccines using a simulation model to project these outcomes up to 20 years after vaccination.

### Study population and timeframe

We included children aged 5 years or younger from 102 low-income, lower-middle-income, and upper-middle-income countries according to World Bank classification (appendix p 5). Included countries must have had at least one estimated *Shigella*-attributable death annually according to the 2019 Global Burden of Disease Study (GBD)^17^ and available economic information. National estimates were aggregated by eligibility for Gavi, the Vaccine Alliance; World Bank income classification; and by WHO region to assess regional trends (African region, region of the Americas, Eastern Mediterranean region, South-East Asia region, and Western Pacific region). Countries’ Gavi eligibility status was based on 2020 criteria.^18^ Population estimates were based on UN Population Division country estimates and projections from 2025 to 2044.^19^

### *Shigella* morbidity and mortality burden

We modelled *Shigella* morbidity and mortality burden in children aged 5 years or younger. Burden estimates depended upon *Shigella*-attributable proportions of diarrhoeal episodes and deaths from acute diarrhoea and deaths from other infections related to *Shigella*-attributable stunting (appendix p 1).^6^ Diarrhoeal morbidity was based on episodes per child from a systematic review.^20^ Mortality estimates for diarrhoeal disease were based on 2019 GBD estimates.^17^ We calculated *Shigella*-attributable deaths and diarrhoeal episodes (and cause-agnostic diarrhoeal episodes) and projected morbidity and mortality estimates from 2019 to 2044 (appendix p 2) using WHO region-specific aetiological fractions of *Shigella*-attributed morbidity and mortality.^17^ Further, we used fractions of episodes considered to be less severe diarrhoea and moderate-to-severe diarrhoea after visits to health-care facilities from the Global Enteric Multicenter Study (GEMS) 1A follow-on study^9^ in our estimates (appendix p 1).

### Effects of *Shigella*-attributable stunting

We applied our previous methods^6^ to determine *Shigella*-attributable stunting, with some modifications (appendix p 12). First, we calculated the shift in child HAZ scores associated with episodes of *Shigella*-related less severe diarrhoea and moderate-to-severe diarrhoea using 2019 GEMS 1A follow-on study results^9^ (table; appendix p 1). In the absence of reliable community-level estimates for diarrhoea treatment, we assumed that, of diarrhoeal episodes when care was sought at a health facility, 25% were moderate-to-severe diarrhoea and 75% were less severe diarrhoea.^9^ Using data from the most recent Demographic and Health Surveys, we estimated the proportion of childhood diarrhoeal episodes for which care was sought by taking the mean proportion across all model countries reporting this variable and substituting the corresponding WHO regional average for those without a country-level estimate (table; appendix p 1). Using our previous approach,^6^ we estimated child mortality from infections for which stunting is a risk factor (eg, lower respiratory infections, malaria, measles, and other-cause diarrhoea; appendix p 2).^1^

**Table:** Model inputs and parameters for best-case scenario and ranges used in uncertainty and sensitivity analyses

	**Values**	**Sensitivity range**	**Distribution**	**Source**
**Burden**				
Population estimates	Varies by country	..	NA	UNDP 2025-45^19^
Diarrhoeal mortality for children aged ≤5 years	Varies by country	10%	Log normal	GBD 2019^17^
Diarrhoeal mortality rate of change (2025–44)	Varies by region and income classification	25%	Normal	..
Other infectious disease mortality for children aged ≤5 years	Varies by country	..	Log normal	GBD 2019^17^
Other infectious disease mortality rate of change (2025–44)	Varies by region and income classification	..	Normal	..
Annual rate of diarrhoeal episodes in children aged ≤5 years (95% UI)	African region 3·3 (2·1–5·0); region of the Americas 3·2 (2·6–3·8); Eastern Mediterranean region 2·9 (1·6–4·2); European region 2·8 (2·3–3·3); South-East Asia region 2·4 (1·5–3·3); and Western Pacific region: 2·2 (1·3–2·5)	25%	Log normal	Anderson et al^6^ and Walker et al^21^
Diarrhoeal episodes annual rate of decline (2025–44)	0·37% (SD 0·09%)	25%	Normal	GBD 2019^17^
Aetiological fraction of *Shigella*-attributable mortality by WHO region (95% UI)	African region 22·4% (9·6–40·3); region of the Americas 13·4% (5·4–26·8%); Eastern Mediterranean region 11·7% (4·2–24·6%); European region 10·2% (3·8–21·7%); South-East Asia region 9·0% (3·4–19·5%); and Western Pacific region 11·2% (4·4–22·5%)	50%	Beta	GBD 2019^17^
Aetiological fraction of *Shigella*-attributable morbidity by WHO region (95% UI)	African region 16·6% (9·1–27·7%); region of the Americas; 12·6% (6·9–20·4%); Eastern Mediterranean region 10·8% (5·8–18·5%); European region 6·8% (3·7–11·3%); South-East Asia region 8·2% (4·5–13·6%); and Western Pacific region 7·5% (4·1–12·2%)	50%	Beta	GBD 2019^17^
Moderate and severe stunting estimates	Varies by country	..	..	DHS and JME^22^
Annual stunting rate of decline	African region 1·7%; region of the Americas 2·6%; Eastern Mediterranean region 1·5%; European region 3·7%; South-East Asia region 2·3%; and Western Pacific region 1·6%	..	..	JME^22^
*Shigella*-attributable stunting cases (by episodes of moderate-to-severe diarrhoea)	0·072 (SD 0·017) shift in HAZ	25%	Normal	GEMS 1A^9^
*Shigella*-attributable stunting cases (by episodes of less severe diarrhoea)	0·052 (SD 0·018) shift in HAZ	25%	Normal	GEMS 1A^9^
Percentage of *Shigella*-attributable episodes of moderate-to-severe diarrhoea	25% of children who sought care at a health-care facility	..	NA	GEMS 1A^9^
Percentage of *Shigella*-attributable episodes of less severe diarrhoea	75% of children who sought care at a health-care facility	..	NA	GEMS 1A^9^
Percentage of caretakers who sought care at a health facility after a child's diarrhoeal episode (95% UI)	57% (55–59)	..	Beta	DHS data
**Vaccination inputs and medical costs**				
*Shigella* vaccine effectiveness against mortality and episodes of moderate-to-severe diarrhoea (95% UI)	60% (40–80)	33%	Beta	iTPP^23^ assumption
*Shigella* vaccine effectiveness against episodes of less severe diarrhoea (95% UI)	40% (20–60)	33%	Beta	iTPP^23^ assumption
Gavi-eligible dose price (95% UI)	$2·00 (1·00–3·00)	50%	Gamma	iTPP^23^ assumption
Gavi-ineligible dose price in lower-middle-income countries (95% UI)	$7·10 (3·55–10·65)	50%	Gamma	MI4A^24^ analysis
Gavi-ineligible dose price in upper-middle-income countries (95% UI)	$9·73 (4·87–14·60)	50%	Gamma	MI4A^24^ analysis
Gavi-ineligible dose price in region of the Americas (95% UI)	$6·75 (3·73–10·12)	50%	Gamma	MI4A^24^ analysis
Doses for full course	2	..	NA	iTPP^23^ assumption
Administration cost	$1·37 in low-income countries, $2·05 in lower-middle-income countries, and $2·05 in upper-middle-income countries	40%	Gamma	Debellut et al^25^
Cost of *Shigella* illness	Varies by country; outpatient median $7·08 per episode and inpatient mean $71·48 per episode	40%	Gamma	Baral et al^26^
Inpatient visit rate (95% UI)	African region 2·2% (1·7–2·5); region of the Americas 1·9% (1·8–2·0); Eastern Mediterranean region 2·1% (1·7–2·8); European region 1·6% (1·5–1·7); South-East Asia region 2·2% (1·9–2·6); and Western Pacific region 2·3% (2·2–2·6)	..	Beta	Debellut et al^25^ and Walker et al^21^
Outpatient visit rate (cases taken to health-care facility; 95% UI)	57% (55–59) of *Shigella* cases sought treatment annually	..	Beta	DHS data

All costs are presented in 2019 US$. DHS=Demographic and Health Surveys. GBD=Global Burden of Disease Study. GEMS=Global Enteric Multicenter Study. HAZ=height-for-age Z score. iTPP=Intervention Target Product Profile. JME=Joint Child Malnutrition Estimates. MI4A=Market Information for Access to Vaccines. NA=not applicable. UI=uncertainty interval.

### Burden outcome measures

Burden outcome measures included episodes of *Shigella*-attributable moderate-to-severe diarrhoea and less severe diarrhoea, *Shigella*-attributable direct mortality, stunting attributable to *Shigella*-related moderate-to-severe diarrhoea and less severe diarrhoea, stunting-related deaths from other infections, and disability-adjusted life-years (DALYs). Although childhood stunting is a risk factor associated with other infection-related deaths, it is not directly included in DALY calculations. All mortality outcomes, including stunting-related deaths from other infections, were translated to DALYs^27^ that were not age-weighted but were discounted 3% annually from 2025 to 2044 (appendix p 1). We calculated all outcomes annually and cumulatively from vaccine introduction.

### Vaccine-related assumptions and inputs

We assessed the effect of the national introduction of *Shigella* standalone vaccine candidates administered to children after age 6 months, assuming a 2025 introduction by all countries. We examined 20 annual birth cohorts of children over the first 5 years of life (following each cohort until 2044). The vaccine was assumed to be 60% effective^23^, ^28^ in preventing deaths and episodes attributable to *Shigella*-related moderate-to-severe diarrhoea and 40% effective in preventing deaths and episodes attributable to *Shigella*-related less severe diarrhoea (table). Additionally, we assumed that protection is conferred after the second dose, with no protection for partially vaccinated children; that effectiveness does not wane during the first 5 years after immunisation; and that herd protection is not conferred by vaccination. We did not include vaccine effectiveness after age 5 years. We calculated vaccination coverage using country-specific 2020 coverage estimates for the third dose of diphtheria-pertussis-tetanus vaccine (DPT3; DPT2 coverage not reported^29^). In countries with estimates of below 90%, we projected vaccination coverage as improving 1% per year up to 90%.

As vaccine price is uncertain, we used a 2021 *Shigella* vaccine product profile^23^ to inform our base price (US$2·00/dose) for Gavi-eligible countries, exploring prices ranging from $1·00 to $3·00 per dose. For lower-middle-income and upper-middle-income countries that were ineligible for Gavi in 2020, we used vaccine market data from the WHO Market Information for Access to Vaccines^24^ to estimate vaccine prices. However, we calculated separate prices for Gavi-ineligible countries in the region of the Americas regardless of income class because vaccines in this region are procured via the Pan American Health Organization Revolving Fund (table; appendix p 3).

In the absence of published medical costs of *Shigella* diarrhoea for study countries, we used country-specific estimates of direct inpatient and outpatient medical costs associated with moderate-to-severe diarrhoea.^26^ We assumed that patients with severe diarrhoea were referred for inpatient care using published estimates (table).^21^ We assumed that vaccine administration costs were similar to those of rotavirus vaccines.^25^ All costs were adjusted to^30^ and reported in 2019 US$ and were discounted at 3% per year.

### Cost-effectiveness analysis

We used a health-system perspective to calculate cost-effectiveness estimates. Vaccination costs include vaccine administration cost, vaccine price, vaccine demand, and 10% wastage from 2025 to 2044. We calculated net costs for each country and aggregated results by WHO region, World Bank income classification, and Gavi eligibility (appendix p 3). We calculated vaccine benefits for each aggregate over 20 years, using projected benefits for the first 5 years of life for children vaccinated in each annual birth cohort (appendix p 3). We calculated primary cost-effectiveness measures—mean and median incremental cost-effectiveness ratios (ICERs)—by dividing incremental costs associated with vaccine introduction by DALYs averted for each aggregate (appendix p 3). Our comparator scenario was no vaccination.

### Scenario analyses

We calculated three ICERs representing different scenarios: assuming vaccination is effective against moderate-to-severe diarrhoea episodes, mortality, and medical costs attributable to *Shigella* (scenario 1); scenario 1 plus less severe diarrhoea episodes, mortality, and medical costs attributable to *Shigella* (scenario 2); and scenario 2 plus stunting attributable to moderate-to-severe diarrhoea and less severe diarrhoea attributable to *Shigella* and associated mortality from other infections (scenario 3, total *Shigella* burden). We also explored a scenario in which vaccination is only effective against the burden associated with *Shigella-*attributable moderate-to-severe diarrhoea (ie, episodes, mortality, and stunting), which we termed MSD+S only.

### Simulations, uncertainty, and sensitivity analyses

We used Monte Carlo simulation methods to generate model point estimates and uncertainty and sensitivity analyses in R (version 4.1.2). We characterised key input variables as distributions (table) and simulated 1000 replicates. Uncertainty is presented as 95% uncertainty intervals (UIs) representing 2·5% and 97·5% quantile estimates. For ICERs we also present IQRs. We used one-way sensitivity analyses to estimate the effect of changing select input values on ICERS, presented as ranges and percentages of the total variance explained by each input.

### Role of the funding source

The funder of the study had no role in study design, data collection, data analysis, data interpretation, or writing of the report.

## Results

From 2025 to 2044, we estimated that *Shigella* would be associated with 1·6 billion (95% UI 1·0−2·4) episodes of less severe diarrhoea and 543 million (341−804) episodes of moderate-to-severe diarrhoea in children younger than 5 years across all countries. An estimated 109 million (39−204) children would have *Shigella*-attributable stunted growth (appendix p 6). Total deaths from *Shigella*-related diarrhoea in children younger than 5 years without vaccination would be 1·4 million (0·8−2·1) and would cost country health-care systems $11·1 billion (7·5−15·4; appendix p 6).

**Figure 1:**
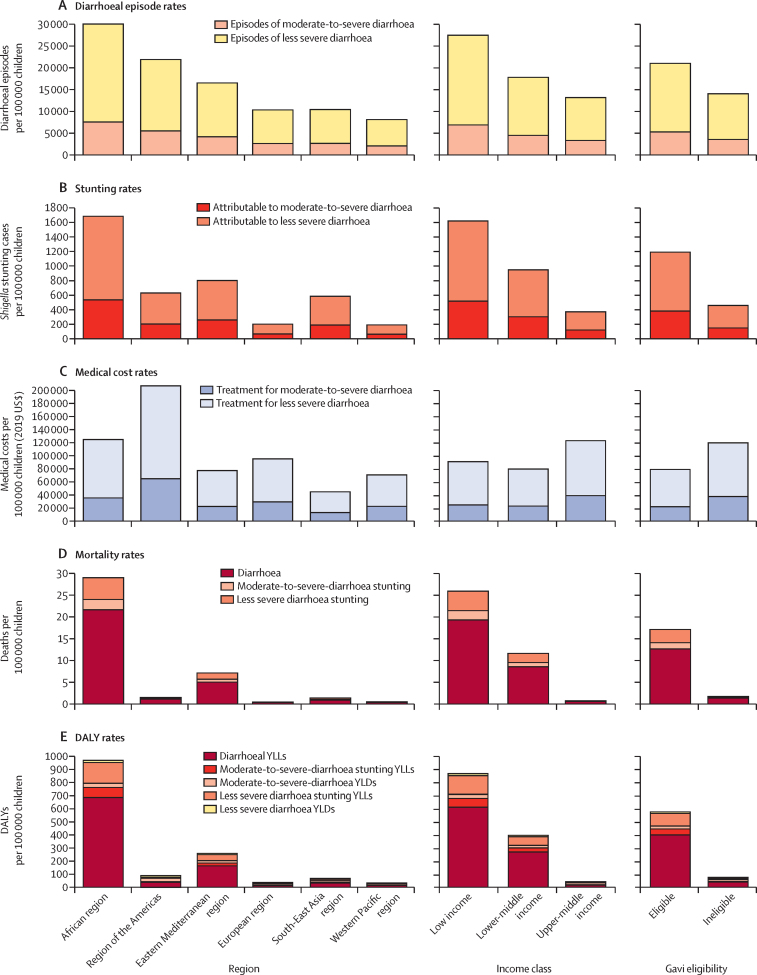
Health and economic burden rates of *Shigella*-attributable diarrhoea and stunting by WHO Region, World Bank income classification, and Gavi eligibility DALY=disability-adjusted life-year. YLD=year lived with disability. YLL=year of life lost.

Our results indicate that, over 20 years, the WHO African region would have the highest estimated *Shigella*-attributable health burden, including the highest burden of episodes of moderate-to-severe diarrhoea and less severe diarrhoea, stunting cases, total mortality, and DALY rates across regions (figure 1; appendix pp 6–10). The African region's burden would be substantially higher than that of other regions, with morbidity rates (*Shigella*-attributable diarrhoeal episodes and stunting cases) twice as high and total *Shigella*-attributable mortality and DALY rates four times as high as those of the Eastern Mediterranean region, the region with the second highest health burden (figure 1; appendix pp 6–10). The region of the Americas would have the highest *Shigella*-attributable medical cost rate, the second highest episode rate of *Shigella*-attributable diarrhoeal episodes (figure 1A, C), but low mortality and DALY rates (figure 1C, D). When assessed by income classification, low-income countries would have the highest *Shigella*-attributable health burden, with the highest rates of episodes of moderate-to-severe diarrhoea and less severe diarrhoea, stunting cases, total mortality, and DALYs (figure 1; appendix p 6), whereas upper-middle-income countries would have the highest medical cost rates (figure 1C). Also, Gavi-eligible countries would have 1·5 times higher diarrhoeal episodic rates, nearly 3 times higher stunting rates, and 10 times higher mortality rates than those of Gavi-ineligible countries (figure 1A, B, D; appendix p 9).

When considering the incremental effect of including the burden of less severe diarrhoea, projected diarrhoeal episodes increased by 75% and stunting cases by 68% (appendix p 6). Including stunting cases attributable to *Shigella-*related less severe diarrhoea increased mortality estimates by 17% (region of the Americas) to 25% (South-East Asia region) in WHO regions, 15% (upper-middle income) to 18% (low income and lower-middle income) in income classes, and 15% (ineligible) to 17% (eligible) by Gavi eligibility status (figure 1D; appendix p 8). Inclusion of years lived with disability from less severe diarrhoea and years of life lost from acute *Shigella* episodes and from other infections due to *Shigella*-attributable stunting increased DALYs by 18% (African region) to 27% (South-East Asia region) in WHO regions, 18% (low income) to 24% (upper-middle income) in income classes, and 19% (eligible) to 20% (ineligible) by Gavi status (figure 1E; appendix p 9). Including medical costs of less severe diarrhoea resulted in increasing costs across WHO regions (2**·**2 times higher in the region of the Americas and Western Pacific region and 2**·**5 times higher in the African, Eastern Mediterranean, and South-East Asia regions), income classes (2·2 times higher in upper-middle-income countries and 2·7 times in low-income countries), and Gavi eligibility status (2·2 times higher in eligible countries and 2·6 times higher in ineligible countries; figure 1C; appendix p 9).

According to our estimates, *Shigella* vaccination would prevent 548 million (95% UI 244−1010) episodes of less severe diarrhoea and 280 million (150−461) episodes of moderate-to-severe diarrhoea across all countries over 20 years (appendix p 6). Vaccination would prevent 43 million (13−92) children from having *Shigella*-attributable stunted growth, 590 000 (297 000–983 000) deaths, and 20 million (11–33) DALYs. Vaccination would avert $4·4 billion (2·2–7·4) in medical costs and result in an ICER (scenario 3) of $849 (423–1575; median $790 [IQR 635–1005]) per DALY averted.

**Figure 2:**
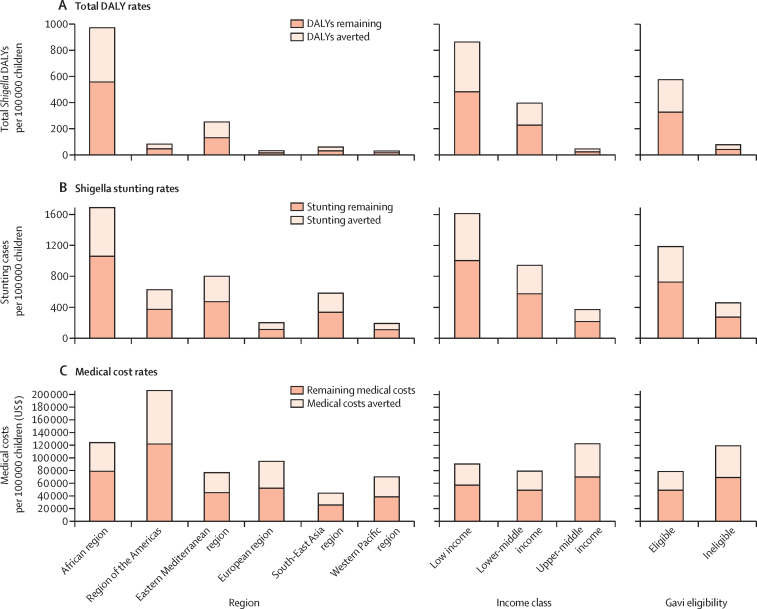
Potential vaccination-averted *Shigella*-attributable health and economic burden rates by WHO region, World Bank income classification, and Gavi eligibility All costs are presented in 2019 US$. DALY=disability-adjusted life-year.

*Shigella* vaccination would avert the greatest amount of DALY burden in the African region—3·4 times that of the Eastern Mediterranean region, the region with the second highest rates of averted DALYs (figure 2A; appendix p 9). Although the African region would have the highest reduction in stunting episode rates, the region of the Americas, the Eastern Mediterranean region, and the South-East Asia region would also have substantial reductions (figure 2B; appendix p 9). The region of the Americas would have the greatest reduction in estimated medical cost rate (figure 2C; appendix p 9). Low-income and Gavi-eligible countries would have the greatest reduction in estimated DALYs and stunting rates (figure 2A, B; appendix p 9), and upper-middle-income and Gavi-ineligible countries would have the greatest reduction in medical costs (figure 2C; appendix p 9).

**Figure 3:**
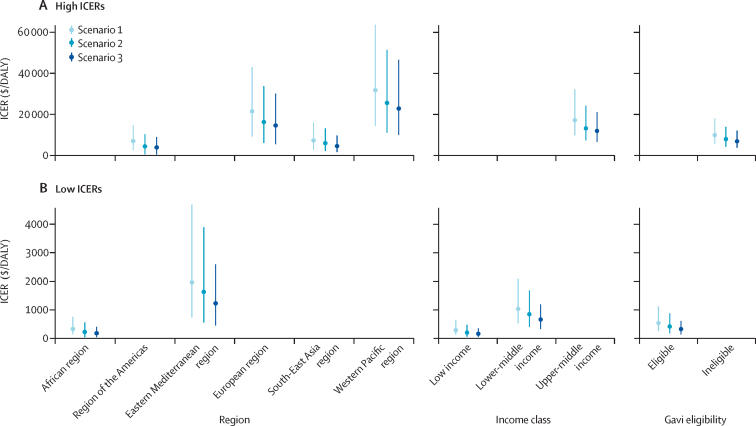
Change in ICERs when including additional burden Scenario 1 ICERs were calculated by including the DALYs associated with episodes of moderate-to-severe diarrhoea (years of life lost and years lived with disability). Scenario 2 ICERs were calculated with DALYs associated with episodes of moderate-to-severe diarrhoea and less severe diarrhoea (years of life lost and years lived with disability). Scenario 3 ICERs were calculated with DALYs associated with episodes of moderate-to-severe diarrhoea and less severe diarrhoea plus stunting attributable to moderate-to-severe diarrhoea and less severe diarrhoea. Lines around points represent 95% uncertainty intervals around ICERs. For ease of visual comprehension, ICERs of 4000 or greater are classified as high and ICERS below 4000 are classified as low. All ICERs are presented in 2019 US$. DALY=disability-adjusted life-year. ICER=incremental cost-effectiveness ratio.

When including the full burden of episodes of *Shigella*-attributable less severe diarrhoea and moderate-to-severe diarrhoea and stunting (scenario 3), vaccination would be most cost-effective (lowest ICERs) in the African region ($161 [95% UI 18–383]; median $145 [IQR 99–206]) and Eastern Mediterranean region ($1210 [434–2575; median $1106 [797–1505]) per DALY averted, low-income ($143 [33–332]; median $129 [92–177]) and lower-middle-income countries ($644 [304–1169]; median $604 [484–752]) per DALY averted; and Gavi-eligible countries ($308 [124–589]; median $285 [219–369]) per DALY averted (figure 3; appendix pp 9–11). The MSD+S only scenario was more cost-effective than scenario 1, but less cost-effective than scenarios 2 and 3 (appendix pp 9–11).

**Figure 4:**
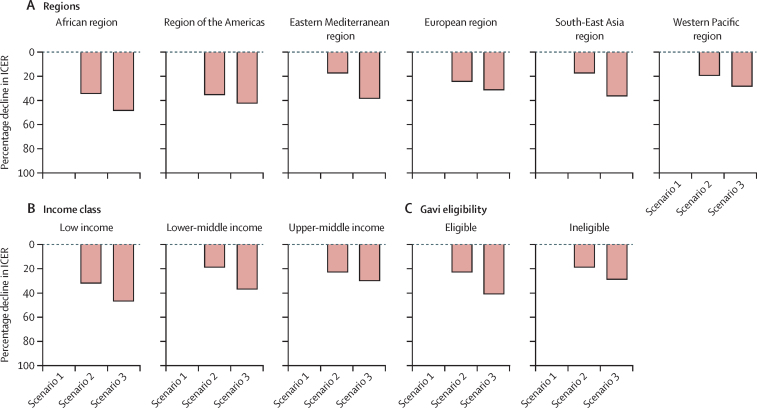
Percentage decline in ICERs when adding episodes of less severe diarrhoea and stunting attributable to moderate-to-severe diarrhoea and less severe diarrhoea to *Shigella* burden Scenario 1 ICERs were calculated by including the DALYs associated with episodes of moderate-to-severe diarrhoea (years of life lost and years lived with disability). Scenario 2 ICERs were calculated with DALYs associated with episodes of moderate-to-severe diarrhoea and less severe diarrhoea (years of life lost and years lived with disability). Scenario 3 ICERs were calculated with DALYs associated with episodes of moderate-to-severe diarrhoea and less severe diarrhoea plus stunting attributable to moderate-to-severe diarrhoea and less severe diarrhoea. DALY=disability-adjusted life-year. ICER=incremental cost-effectiveness ratio.

The addition of less severe diarrhoea episodes attributable to *Shigella* and medical costs (scenario 2) resulted in improved ICERs for the African region (34% reduction), region of the Americas (35% reduction), low-income countries (32% reduction), and Gavi-eligible countries (23% reduction; figure 4). The further inclusion of stunting attributable to *Shigella*-related less severe diarrhoea and moderate-to-severe diarrhoea (percentage difference between scenarios 2 and 3) showed the greatest effect on the mean ICERs of the Eastern Mediterranean region (21% reduction), the South-East Asia region (19% reduction), lower-middle-income countries (18% reduction), and Gavi-eligible countries (18% reduction; figure 4).

**Figure 5:**
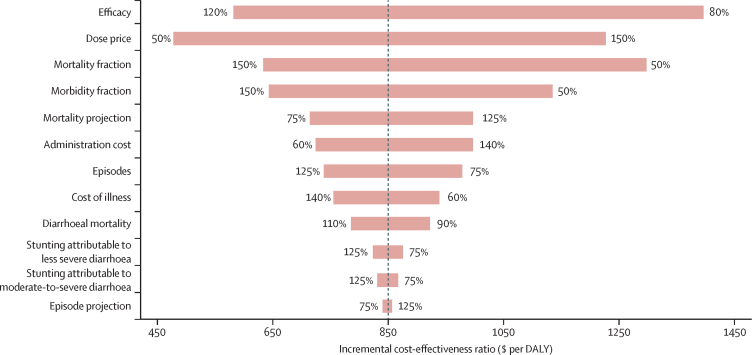
Tornado diagram of one-way probabilistic sensitivity analysis of the key input variables on global *Shigella* vaccination cost-effectiveness in 102 countries from 2025 to 2044 Ranges of variables (listed in the table) are displayed at the ends of the corresponding bars. Mortality fraction and morbidity fraction represent the variation in the fraction of overall *Shigella*-attributable diarrhoeal mortality or morbidity. Mortality projection and episode projection are the variations in the rates of diarrhoeal mortality and episodes projected from 2025 to 2044. Stunting attributable to less severe diarrhoea or moderate-to-severe diarrhoea refers to the number of other infectious disease deaths caused by stunting attributable to *Shigella*-related less severe diarrhoea and moderate-to-severe diarrhoea. All ICERs are presented in 2019 US$. All burden estimates are without vaccination. DALY=disability-adjusted life-year. ICER=incremental cost-effectiveness ratio.

According to the ICER one-way sensitivity analysis for all countries, four variables accounted for 88% of the variance (figure 5). The scenario 3 ICER ($849 [95% UI 423–1575; median $790 [IQR635–1005] per DALY averted) was most sensitive to changes in vaccine efficacy (31% of variance), vaccine price (26%), *Shigella*-attributable mortality (20%), and *Shigella*-attributable morbidity (11%). Mortality projections accounted for 3% of variance, administrative costs accounted for 4%, and cost of illness accounted for 2%. Remaining variables accounted for 1% or less.

## Discussion

Based on the vaccine efficacy assumptions outlined in the target product profiles, our model indicates that *Shigella* vaccination could potentially prevent a third of episodes of less severe diarrhoea and stunting cases attributable to *Shigella*, half of moderate-to-severe diarrhoea episodes attributable to *Shigella*, and almost half of *Shigella*-attributable total mortality and DALY burden across all countries over 20 years. These results show that a potential *Shigella* vaccine could have a considerable public health benefit, even at a fairly modest range of effectiveness explored in our model. Our estimates indicate that *Shigella* vaccination would avert the most mortality and DALY burden in groups with the highest *Shigella*-attributable health burden—the African region, Eastern Mediterranean region, low-income countries, and Gavi-eligible countries. If *Shigella* vaccination could reduce childhood stunting as simulated in our model, the African region, Eastern Mediterranean region, South-East Asia region, and low-income countries would benefit the most. The region of the Americas, upper-middle-income countries, and Gavi-ineligible countries had the largest economic burden and, accordingly, medical costs averted by vaccination. When compared with *Shigella*-attributable moderate-to-severe diarrhoea only (scenario 1), ICER estimates decreased by 17% upon inclusion of episodes of less severe diarrhoea (scenario 2) and 36% upon inclusion of stunting attributable to moderate-to-severe diarrhoea and less severe diarrhoea (scenario 3) across all countries. Including medical costs associated with less severe diarrhoea doubled economic burden, resulting in substantial improvement in ICERs, especially for high-burden regions and low-income and Gavi-eligible countries. Vaccination was most cost-effective in the African region, low-income countries, and Gavi-eligible countries across all scenarios.

When including *Shigella*-attributable episodes of less severe diarrhoea (scenario 2 *vs* scenario 1) in ICERs, cost-effectiveness improved most for the region of the Americas because of high rates of less severe diarrhoea medical costs, all diarrhoea episodes, and *Shigella*-attributable aetiological fractions, although the mean ICER remained high (>$4700). The inclusion of stunting associated with *Shigella*-attributable moderate-to-severe and less severe diarrhoea (scenario 3 *vs* scenario 2) in ICERs resulted in the Eastern Mediterranean region, South-East Asia region, low-income countries, and lower-middle income-countries had the greatest improvement, stemming from high mortality from stunting associated with *Shigella*-attributable less severe diarrhoea. These results indicate that, although vaccination might not have a substantial short-term benefit for certain countries or regions, if the vaccine can reduce childhood stunting and its downstream effects, areas with considerable childhood stunting might experience long-term benefits.

When including the burden attributable to less severe diarrhoea, *Shigella* vaccination is still less cost-effective than malaria vaccination (ICERs of $80−87 in African settings),^31^ but is approaching cost-effectiveness of pneumococcal^32^ and live oral rotavirus vaccination for certain regions.^25^ For example, an analysis of rotavirus vaccination reported ICERs of $108 for the African region and $264 for Gavi-eligible countries,^25^ and we report means of $161 for the African region and $308 for Gavi-eligible countries. However, ICERs for other regions were considerably higher than those for rotavirus. Despite this finding, *Shigella* vaccination should still be considered for introduction in high-burden areas.

The sensitivity analysis showed that the two most influential inputs were vaccine efficacy and vaccine price, both of which are still unknown. Ongoing clinical trials (NCT05073003, NCT04602975, NCT04634513, and NCT04242264) will provide key information on vaccine efficacy, dose schedule, timing, and combination potential, influencing future vaccine impact estimates. *Shigella* vaccine efficacy, which is currently unknown, probably differs against moderate-to-severe diarrhoea and less severe diarrhoea and affects the estimates of averted medical costs and stunting. The next two most influential inputs were the aetiological fractions of *Shigella*-attributable diarrhoeal mortality and morbidity.

Comparing *Shigella* aetiological fraction estimates used here to those used in our previous burden model provides additional insight into their importance. Previously, we used culture-based aetiological fractions adjusted for molecular sensitivity,^6^ resulting in the Eastern Mediterranean region having the highest *Shigella* mortality rate and the lowest ICERs.^7^ By contrast, 2019 GBD *Shigella* aetiological fraction estimates were highest for the African region and considerably higher for the European region, South-East Asia region, and Western Pacific region than in our previous model iteration.^6^ As a result, *Shigella* vaccination was most cost-effective in the African region and more cost-effective in the European region, South-East Asia region, and Western Pacific region compared with in previous estimates.

Although our results are based on the best available data and empirical evidence, several limitations remain. An underlying assumption is that *Shigella* infection and disease results in childhood stunting. Although large multisite and other studies have linked *Shigella*-attributable disease to growth impacts^10^, ^11^, ^13^ and inflammatory markers suspected to mediate this relationship,^12^, ^14^ the underlying mechanism between the two remains an area of ongoing research. Many aspects of this relationship remain largely uncharacterised—the most notable being whether a vaccine can ameliorate enteric infection-related growth impacts. Multiple *Shigella* vaccines are in preclinical studies and phase 1 or phase 2 clinical trials; however, trials in young children are pending. Although our results show the potential ability of *Shigella* vaccination to avert stunting, once such a vaccine is developed the crucial next step will be vaccine probe studies that include secondary endpoints on prevention of linear growth faltering and childhood stunting.^33^ Also, although we include the burden of less severe diarrhoea that is based on the findings of one study,^9^ other studies^10^, ^34^ have shown an association between subclinical *Shigella* infections and growth faltering, indicating that the inclusion of less severe diarrhoea is conservative. As the contribution of subclinical infections to linear growth faltering is better characterised, future burden models should integrate the effects of subclinical *Shigella* infections on childhood linear growth faltering and stunting. Details of the delivery schedule will be important for future cost and impact estimates but are currently not known.

Our estimates have wide UIs, reflecting uncertainty of inputs and assumptions. Aetiological fractions were based on GBD 2019 data and limited by data availability issues in neonatal age groups and some geographical regions.^15^ To improve the accuracy of estimates of the *Shigella* health and economic burden, future models should use region-representative episode prevalence and associated medical costs from community surveillance data. We used a comparator of no vaccination—other comparators could be examined as described in our companion analysis.^33^ Including other secondary benefits of vaccination, such as herd immunity and effects on antibiotic resistance, will also improve future estimates.

Our companion analysis shows the importance of including the long-term economic benefits of *Shigella* vaccination and macroeconomic implications of this relationship.^33^ Including future gains in adult productivity among vaccination benefits resulted in the vaccine being cost-effective in almost all regions or country groupings studied. Future economic analyses from the societal perspective would provide a necessary, more comprehensive understanding of *Shigella* burden.

This study builds on our previous analysis, which focused on the effects of reducing the burden of moderate-to-severe diarrhoea. Our model improves our cost-effectiveness projections by accounting for the burden of less severe diarrhoea attributable to *Shigella*. Our results show the importance of assessing the full burden of disease and how doing so might uncover patterns or factors to consider when developing vaccines and planning implementation. Our findings also enhance the estimated value of *Shigella* vaccination, which is important as policy makers consider a multiplicity of other public health interventions.^35^ These findings require assessment by large-scale vaccine trials in populations at high risk, ideally showing that non-acute health effects, such as linear growth faltering and stunting, even from less severe disease, can be ameliorated by vaccination**.**

## Data sharing

Data for this study were taken from publicly available sources that are documented in this Article. Individual participant data were not used in this research. The model has been described in sufficient detail in the Article and appendix. There are no plans to make the model publicly available.

For the **Demographic and Health Surveys** see https://www.statcompiler.com/en

## Declaration of interests

We declare no competing interests.
