# Supplementary material for: Potential impact and cost-effectiveness of Shigella vaccination in 102 low-income and middle-income countries in children aged 5 years or younger: a modelling study
Source: Lancet Glob Health. 2023 May 16;11(6):e880–91. doi: 10.1016/S2214-109X(23)00192-4 (PMC10206199; doi:10.1016/S2214-109X(23)00192-4)

THE LANCET
Global Health

Supplementary appendix

This appendix formed part of the original submission and has been peer reviewed. We post it as supplied by the authors.

Supplement to: Anderson JD IV, Bagamian KH, Pecenka CJ, et al. Potential impact and cost-effectiveness of *Shigella* vaccination in 102 low-income and middle-income countries in children aged 5 years or younger: a modelling study. *Lancet Glob Health* 2023; **11:** e880–91.

Supplementary Materials

Table of Contents

Diarrhoea mortality and morbidity 1

Mortality Projections 2

Cost-effectiveness analysis 2

References 3

Supplementary Table 1 5

Supplementary Table 2 6

Supplementary Table 3 9

Supplementary Table 4 11

Supplementary Figure 1 12

Diarrhoea mortality and morbidity

Our aetiological estimates are based on IHME estimates for both mortality and morbidity.[1] We assumed the aetiological fraction for Years Lived with Disability (YLD) as a proxy for diarrhoeal episodes. We assumed that aetiology does not differ between the causes of less severe or moderate-to-severe episodes and mortality. *Shigella*-attributed diarrhoeal episodes were estimated by multiplying the aetiological fractions for each World Health Organization (WHO) region by regional diarrhoeal disease incidence from a systematic review (clinically relevant diarrhoea with three or more loose or liquid stools in a 24-hour period).[2]

YLDs that were used in the calculation Disability-Adjusted Life Years (DALYs) for children under five were calculated using durations of 4.3 days (0.01 years) for mild diarrohea and 8.4 days (0.02 years) for moderate-to-severe diarrhoea,[3] Disability weights of 0.074 mild diarrhoea and 0.2175 for moderate-to-severe diarrhoea were based on 2019 Global Burden of Disease weights.[4] DALYs were not age-weighted but were discounted 3% annually from 2025−2044. Years of Life Lost was based on a life expectancy of 84 years, based on 2020 life expectancy in Japan.[5]

The potential increase in *Shigella*-attributable stunting in children under five was estimated for each country. We used a pooled analysis from Global Enteric Multicenter Study (GEMS) 1A[6] that reported differential height-for-age-z-scores (HAZ) shifts caused by stunting cases for children by severity of diarrhoea. We calculated the mean fraction of moderate-to-severe diarrhoea (MSD) and less severe diarrhoea (LSD) cases occurring in three age groups (<12, 12–23, and 24–59 months) and applied the mean HAZ differences (between cases and controls) for MSD and LSD to the corresponding age group. The average shift was 0·072 HAZ for MSD cases and 0·054 HAZ for LSD cases. We assumed this shift impacts the entire population, not just the most at-risk children.[7] This output was used to calculate fractions of moderate and severe stunting attributed to *Shigella* disease. Then, relative risks of death due to other infectious diseases (ID) was attributed to Shigella-stunting in each country population.

We assumed 75% and 25% of all diarrhoeal episodes where care was sought at a health facility were LSD and MSD, respectively.[6] We adjusted this value by calculating the proportion of children who experienced diarrhoea two weeks prior to the survey[8] and whose caretakers sought care for their diarrhoea at a health facility using DHS data. When

DHS healthcare seeking estimates were not available, we substituted the mean percentage of healthcare seeking of the other countries in the same WHO region. Our analysis showed that 57% of caretakers visit a health facility after a child experienced a diarrhoeal episode (Table 2). Thus, we assumed 14% (25% x 57%) of diarrhoeal episodes were moderate-to-severe and 43% (25% x 57%) of diarrhoeal episodes were less severe within each region.

We estimated the effect of this marginal shift in HAZ from LSD and MSD on the fraction of children who are categorized as moderately and severely stunted. We used recent DHS8, WHO9 and World Bank10 national estimates of the proportion of children classified as moderately stunted (HAZ between -2 and -3 standard deviations less than mean HAZ) and severely stunted (HAZ less than -3 standard deviations less than mean HAZ). We fit the full normal distribution of HAZ ($\boldsymbol{P}$) using national moderately and severe stunting in children estimates as values at -2 and -3 standard deviations below the mean ($\boldsymbol{\mu}$, Eq 1·1) and then calculated two, hypothetical distributions ($\boldsymbol{P'}$) where means are shifted (Δ) by 0·072 (MSD) or 0·054 (LSD) to the right ($\boldsymbol{\mu'}$, Eq. 1·2), simulating a distribution without the effects of MSD episodes on stunting for each pathogen. While Shigella episodes occur among specific children, this approach simulates an average expected shift among all children. We calculated the differences in probabilities of moderate ($\boldsymbol{Y_m}$) and severe stunting ($\boldsymbol{Y_s}$) between the two distributions in Equations 1·3 & 1·4, using the cumulative distribution function. We could not simulate random draws for moderate (≤2 SDs below median HAZ) and severe stunting (≤3 SDs below median HAZ) distributions as we did not have uncertainty estimates of population mean z-scores.

$$P \sim \mathcal{N}(\mu, \sigma^2) \; where \; \sigma = 1 \qquad \text{(Eq. 1·1)}$$

$$P' \sim \mathcal{N}(\mu', \sigma'^2) \; where \; \mu' = \mu + \Delta \; \textbf{and} \; \sigma' = 1 \qquad \text{(Eq. 1·2)}$$

$$Y_m = [P(X < -2\sigma) - P'(X' < -2\sigma')] \qquad \text{(Eq. 1·3)}$$

$$Y_s = [P(X < -3\sigma) - P'(X' < -3\sigma')] \qquad \text{(Eq. 1·4)}$$

These values are used to estimate the increased percentage and number of children classified as moderately and severely stunted by HAZ.

Moderate and severe stunting are risk factors for mortality due to pneumonia, malaria, measles, and diarrhoeal disease. We used the Population Attributable Risk approach (PAR) to estimate the fraction of ID deaths attributable to excess stunting induced by *Shigella* episodes. The risk factor proportion ($\boldsymbol{P_e}$) was the fraction of children with *Shigella*-induced moderate and severe stunting. We used the relative mortality risk associated with different levels of stunting from Black et al.[11] ($\boldsymbol{RR_e}$, Eq 2). For each of these IDs, we used the PAR equation to estimate the fraction of deaths due to *Shigella*-induced stunting in each country.

$$\boldsymbol{PAR = P_e(RR_e - 1)/[1 + P_e(RR_e - 1)]} \qquad \text{(Eq. 2)}$$

We then combined the PARs[11] with national under-five mortality estimates from GBD[1] for each of these causes to estimate deaths due to diarrhoea, measles, pneumonia, and malaria. To avoid double counting, we applied this approach only to diarrhoeal mortality caused by pathogens other than *Shigella*. We assumed that the increased risk of *Shigella*-induced stunting only occurs among children less than five years old.

Mortality Projections

We projected morbidity based on trends from 2019 GBD estimates of YLDs from diarrhoeal episodes. We used the average rate of change in YLDs from 2010−2019 across all countries from GBD. We projected diarrhoeal and other infectious (lower respiratory, malaria, and measles) mortality using an exponential decline function to calculate annual change in GBD estimates from 2010−2019. Annual mortality rates were region-income class aggregates from country estimates. Most GBD disease mortality estimates declined from 2010−2019, except for rate increases in measles (in 7 region-income class aggregates) and malaria (in 4 region-income class aggregates). For those cases, we held mortality rates constant throughout the 2020–2044 projections.

Cost-effectiveness analysis

We estimated *Shigella* vaccine prices using WHO MI4A (Market Information for Access to Vaccines) Vaccine Market data.[12] First, we calculated the average rotavirus and pneumococcal conjugate vaccine prices for the following groups: LMIs and UMIs by WHO region; PAHO (AMRO) countries; and Gavi-ineligible countries. We calculated separate prices for Gavi-ineligible AMRO countries regardless of income class, as vaccines are procured via the Pan American Health Organization (PAHO) Revolving Fund. We used the ratios of average prices in LMI, UMI, and PAHO Gavi-ineligible countries to Gavi-eligible countries and applied them to our Gavi-ineligible dose price estimates.

We calculated vaccination cost (V) cumulated over the first 20 years (t) post vaccine introduction starting in 2025 for each country (c) based on vaccine administration costs, vaccine price, and quantity (birth cohort times coverage rate with 10% vaccine wastage). We estimated our birth cohorts as the fraction of the under 5 population eligible for vaccination at 6 months. We calculated averted costs (A) based on population, vaccine coverage, efficacy, and access to care and medical costs in each country (c). We calculated net costs (N) for each country and aggregated results by region (r), WB income class (w) and Gavi eligibility (g) as:

$$N_{r,w,g} = \sum_{t=20}^{r,w,g}(V_c - A_c) \qquad \text{(Eq. 3)}$$

We calculated vaccine benefits (B) for each region (r) based on the sum of population, coverage (C), efficacy (E), and DALY burden (D) in each country (c) cumulated over the first 20 years (t) after introduction. We calculated the number of children fully vaccinated each year by multiplying the annual birth cohort by the assumed vaccine coverage. We projected benefits for the first five years of life for children vaccinated in each annual birth cohort.

$$B_{r,w,g} = \sum_{t=20}^{r,w,g}(C_c \cdot E_c \cdot D_c) \qquad \text{(Eq. 4)}$$

Our primary cost-effectiveness measure was the regional Incremental Cost-Effectiveness Ratio ($\text{ICER}_{r,w,g}$), which is the aggregated country-level incremental costs associated with introducing each vaccine divided by aggregated country-level health benefit.

$$\text{ICER}_{r,w,g} = \frac{\sum_{t=20}^{r,w,g} N_{r,w,g}}{\sum_{t=20}^{r,w,g} B_{r,w,g}} \qquad \text{(Eq. 5)}$$

References

1 Global Burden of Disease Collaborative Network. Global Burden of Disease Study 2019 (GBD 2019) Results. Inst. Health Metr. Eval. IHME. 2019. http://ghdx.healthdata.org/gbd-results-tool (accessed Jan 25, 2021).

2 Fischer Walker CL, Sack D, Black RE. Etiology of Diarrhea in Older Children, Adolescents and Adults: A Systematic Review. *PLoS Negl Trop Dis* 2010; **4**: e768.

3 Lamberti LM, Fischer Walker CL, Black RE. Systematic review of diarrhea duration and severity in children and adults in low- and middle-income countries. *BMC Public Health* 2012; **12**. DOI:10.1186/1471-2458-12-276.

4 Vos T, Lim SS, Abbafati C, *et al.* Global burden of 369 diseases and injuries in 204 countries and territories, 1990–2019: a systematic analysis for the Global Burden of Disease Study 2019. *The Lancet* 2020; **396**: 1204–22.

5 World Population Prospects 2019, Online Edition. Rev. 1. 2019. https://population.un.org/wpp/Download/Standard/Population/ (accessed Dec 31, 2021).

6 Kotloff KL, Nasrin D, Blackwelder WC, *et al.* The incidence, aetiology, and adverse clinical consequences of less severe diarrhoeal episodes among infants and children residing in low-income and middle-income countries: a 12-month case-control study as a follow-on to the Global Enteric Multicenter Study (GEMS). *Lancet Glob Health* 2019; **7**: e568–84.

7 Roth DE, Krishna A, Leung M, Shi J, Bassani DG, Barros AJD. Early childhood linear growth faltering in low-income and middle-income countries as a whole-population condition: analysis of 179 Demographic and Health Surveys from 64 countries (1993–2015). *Lancet Glob Health* 2017; **5**: e1249–57.

8 USAID. Demographic and Health Survey (DHS) STATcompiler. 2020; published online May 28. http://www.statcompiler.com/en/ (accessed May 28, 2021).

9 UNICEF, WHO, WB. The UNICEF/WHO/WB Joint Child Malnutrition Estimates (JME). UNICEFWHOWB Jt. Child Malnutrition Estim. JME Group Released New Data 2021. 2021; published online May 6. https://www.who.int/news/item/06-05-2021-the-unicef-who-wb-joint-child-malnutrition-estimates-group-released-new-data-for-2021 (accessed July 20, 2021).

10 The World Bank Group. World Development Indicators Databank [Online]. 2021. https://data.worldbank.org (accessed Jan 13, 2022).

11 Black RE, Allen LH, Bhutta ZA, *et al.* Maternal and child undernutrition: global and regional exposures and health consequences. *Lancet* 2008; **371**. DOI:10.1016/s0140-6736(07)61690-0.

12 WHO. Market Information for Access to Vaccines (MI4A). Immun. Vaccines Biol. 2020. https://www.who.int/teams/immunization-vaccines-and-biologicals/vaccine-access/mi4a/mi4a-vaccine-purchase-data (accessed Jan 20, 2022).

13 Anderson JD, Bagamian KH, Muhib F, *et al.* Burden of enterotoxigenic Escherichia coli and shigella non-fatal diarrhoeal infections in 79 low-income and lower middle-income countries: a modelling analysis. *Lancet Glob Health* 2019; **7**: e321–30.

14 ICF. STATcompiler. DHS Program STATcompiler Funded USAID. 2021. https://www.statcompiler.com/en/ (accessed July 14, 2021).

15 Global Burden of Disease Collaborative Network. Global Burden of Disease Study 2019 (GBD 2019) Results. Seattle, United States: Institute for Health Metrics and Evaluation (IHME), 2019. http://ghdx.healthdata.org/gbd-results-tool (accessed Oct 9, 2021).

Supplementary Table 1. The 102 model countries by WHO region, World Bank income classification[10], and Gavi eligibility. *AFRO: African region, AMRO: Region of the Americas, EMRO: Eastern Mediterranean Region, SEARO: Southeast Asian Region, WPRO: Western Pacific Region, LI: Low Income, LMI: Low Middle Income, UMI: Upper Middle Income. Countries in bold italic type are Gavi-eligible.*

Income class	AFRO		AMRO	EMRO	EURO	SEARO	WPRO
**LI**	***Burkina Faso***<br>***Burundi***<br>***Central African Republic***<br>***Chad***<br>***Congo, Dem. Rep.***<br>***Eritrea***<br>***Ethiopia***<br>***The Gambia***<br>***Guinea***<br>***Guinea-Bissau***	***Liberia***<br>***Madagascar***<br>***Malawi***<br>***Mali***<br>***Mozambique***<br>***Niger***<br>***Rwanda***<br>***Sierra Leone***<br>***Togo***<br>***Uganda***	***Haiti***	***Afghanistan***<br>***Somalia***<br>***Sudan***<br>***Yemen***	***Tajikistan***		
**LMI**	Algeria<br>Angola<br>***Benin***<br>Cabo Verde<br>***Cameroon***<br>***Comoros***<br>***Congo, Rep.***<br>***Cote d'Ivoire***<br>Eswatini	***Ghana***<br>***Kenya***<br>***Lesotho***<br>***Mauritania***<br>***Nigeria***<br>***Senegal***<br>***Tanzania***<br>***Zambia***<br>***Zimbabwe***	Bolivia<br>El Salvador<br>Honduras<br>***Nicaragua***	***Djibouti***<br>Egypt, Arab Rep.<br>Morocco<br>***Pakistan***<br>Tunisia	***Kyrgyz Republic***<br>Ukraine<br>***Uzbekistan***	***Bangladesh***<br>Bhutan<br>***India***<br>***Myanmar***<br>***Nepal***<br>Sri Lanka<br>Timor-Leste	***Cambodia***<br>Kiribati<br>***Lao PDR***<br>***Mongolia***<br>***Papua New Guinea***<br>Philippines<br>***Solomon Islands***<br>Vanuatu<br>Vietnam
**UMI**	Botswana<br>Gabon<br>Namibia<br>South Africa		Argentina<br>Brazil<br>Colombia<br>Costa Rica<br>Dominican Republic<br>Ecuador<br>Guatemala<br>Jamaica<br>Mexico<br>Paraguay<br>Peru<br>Suriname	Iraq<br>Jordan	Armenia<br>Azerbaijan<br>Belarus<br>Bulgaria<br>Georgia<br>Kazakhstan<br>Turkey	Indonesia<br>Thailand	China<br>Fiji<br>Malaysia

Supplementary Table 2. Estimated *Shigella*-attributable mortality and morbidity burden without vaccination and burden averted by potential *Shigella* vaccination in children under 5 years of age. Estimates are presented as totals for 102 countries aggregated by WHO region, income classification, Gavi-eligibility and in all 102 countries. Simulation model estimates are presented as mean estimates projected from 2025 (year of introduction) to 2044. All costs are presented in 2019 US$. Results from uncertainty analysis are presented as 95% uncertainty intervals in parentheses below model estimates. *LSD: less severe diarrhoea−MSD: moderate−to−severe−diarrhoea−LI: lower income−LMI: lower middle income−UMI: upper middle income−children: children under five years of age.*

	REGION						INCOME CLASS			GAVI		ALL COUNTRIES
	AFRO	AMRO	EMRO	EURO	SEARO	WPRO	LI	LMI	UMI	Eligible	Ineligible	
NUMBER OF COUNTRIES	42	17	11	11	9	12	26	46	30	53	49	102
LSD episodes (millions)	921	138	182	25	246	115	487	827	314	1,181	446	1,628
	(387.2–1,624.3)	(72.4–231.4)	(78.3–332.5)	(12.7–42.5)	(113.8–453.1)	(56.0–192.7)	(235.4–842.0)	(502.9–1,210.4)	(229.1–422.0)	(661.2–1,864.0)	(328.7–591.0)	(1,023.6–2,412.9)
LSD episodes averted (millions)	294	47	64	10	89	43	157	277	114	389	159	548
	(97.3–632.6)	(19.0–88.1)	(20.2–139.9)	(3.7–19.4)	(32.6–192.5)	(16.4–87.1)	(57.5–336.2)	(122.9–513.5)	(56.2–190.2)	(163.1–763.2)	(78.5–271.0)	(243.6–1,010.4)
MSD episodes (millions)	307	46	61	8	82	38	162	276	105	394	149	543
	(129.1–541.4)	(24.1–77.1)	(26.1–110.8)	(4.2–14.2)	(37.9–151.0)	(18.7–64.2)	(78.5–280.7)	(167.6–403.5)	(76.4–140.7)	(220.4–621.3)	(109.6–197.0)	(341.2–804.3)
MSD episodes averted (millions)	150	24	33	5	46	22	80	142	58	198	82	280
	(56.6–284.2)	(11.7–43.8)	(12.8–66.2)	(2.2–9.2)	(18.2–92.1)	(9.4–40.3)	(34.4–144.2)	(73.7–235.1)	(33.4–87.4)	(97.0–340.4)	(47.8–120.7)	(149.8–461.1)
LSD-attributable stunting episodes (millions)	47	4	8	0	13	2	26	40	8	61	13	74
	(11.7–102.6)	(1.1–7.3)	(2.1–17.9)	(0.1–0.9)	(3.2–27.9)	(0.7–5.2)	(7.0–53.9)	(11.7–76.8)	(2.7–14.7)	(18.1–118.8)	(4.4–23.8)	(22.8–141.2)
LSD-attributable stunting episodes averted (millions)	15	1	3	0	5	1	8	13	3	20	5	25
	(3.1–38.9)	(0.4–2.8)	(0.6–7.0)	(>0.1–0.4)	(1.1–11.1)	(0.2–2.2)	(1.9–22.1)	(3.3–30.7)	(0.9–6.1)	(4.7–48.3)	(1.3–9.8)	(6.1–57.9)
MSD-attributable stunting episodes (millions)	22	2	4	0	6	1	12	19	4	29	6	35
	(8.0–45.9)	(0.7–3.2)	(1.3–7.8)	(>0.1–0.4)	(2.2–12.2)	(0.4–2.2)	(4.8–24.5)	(8.8–33.5)	(1.9–6.2)	(12.5–52.9)	(3.1–10.2)	(16.3–63.1)
MSD-attributable stunting episodes averted (millions)	11	1	2	0	3	1	6	10	2	15	3	18
	(3.5–23.2)	(0.3–1.8)	(0.6–4.6)	(>0.1–0.3)	(1.1–7.4)	(0.2–1.3)	(2.0–12.5)	(3.8–18.5)	(0.9–3.7)	(5.3–28.5)	(1.4–5.9)	(7.0–34.0)
LSD-attributable stunting deaths (1000s)	205	2	21	>0.1	10	2	107	130	3	229	11	240
	(50.6–436.9)	(>0.1–3.9)	(5.7–46.0)	(>0.1–>0.1)	(2.5–21.9)	(>0.1–3.5)	(28.4–218.8)	(35.9–256.8)	(1.1–6.3)	(62.2–461.9)	(3.4–20.8)	(66.3–482.7)
LSD-attributable stunting deaths averted (1000s)	61	>0.1	7	>0.1	4	>0.1	33	39	1	70	3	73
	(12.9–152.5)	(>0.1–1.3)	(1.6–17.0)	(>0.1–>0.1)	(>0.1–8.7)	(>0.1–1.2)	(7.4–82.2)	(9.0–91.5)	(>0.1–2.5)	(15.8–169.2)	(>0.1–7.7)	(16.7–176.5)
MSD-attributable stunting deaths (1000s)	97	>0.1	10	>0.1	5	>0.1	51	62	2	108	5	114
	(34.8–200.1)	(>0.1–1.7)	(3.4–20.6)	(>0.1–>0.1)	(1.7–10.0)	(>0.1–1.5)	(19.2–99.8)	(25.2–117.9)	(>0.1–2.7)	(43.2–214.9)	(2.5–9.2)	(45.9–224.6)
MSD-attributable stunting deaths averted (1000s)*	44	>0.1	5	>0.1	3	>0.1	24	28	>0.1	50	3	53
	(13.9–93.6)	(>0.1–>0.1)	(1.6–11.5)	(>0.1–>0.1)	(>0.1–5.8)	(>0.1–>0.1)	(8.0–48.8)	(9.4–55.2)	(>0.1–1.7)	(17.2–100.9)	(1.0–4.7)	(18.1–105.1)

	REGION						INCOME CLASS			GAVI		ALL COUNTRIES
	AFRO	AMRO	EMRO	EURO	SEARO	WPRO	LI	LMI	UMI	Eligible	Ineligible	
Total deaths (1000s)*	1,189	12	105	1	43	9	614	722	23	1,287	72	1,359
	(620.8–1,912.8)	(5.8–22.4)	(49.2–184.3)	(>0.1–2.7)	(20.1–74.5)	(4.5–16.5)	(337.4–968.9)	(415.1–1,095.2)	(15.8–32.3)	(718.1–2,004.7)	(49.8–100.8)	(770.3–2,106.1)
Total deaths averted (1000s)*	507	5	51	>0.1	22	4	272	306	12	556	34	590
	(231.5–881.3)	(2.3–10.2)	(21.0–98.2)	(>0.1–1.5)	(9.3–40.9)	(1.8–8.2)	(131.5–463.3)	(156.2–498.1)	(6.7–18.2)	(275.6–933.0)	(19.1–51.1)	(297.0–982.7)
MSD DALYs (1000s)	29,402	503	2,608	70	1,234	385	15,188	17,982	1,032	31,790	2,412	34,202
	(12,454–52,124)	(279–845)	(1,001–5,088)	(39–112)	(572–2,238)	(216–634)	(7,017–26,487)	(9,048–29,447)	(774–1,338)	(15,314–54,006)	(1,732–3,285)	(17,144–57,149)
MSD DALYs averted (1000s)	13,317	245	1,361	41	685	205	7,120	8,165	569	14,627	1,227	15,854
	(5,200–24,873)	(121–423)	(479–2,812)	(19–71)	(273–1,315)	(100–360)	(2,863–13,203)	(3,682–14,004)	(328–841)	(6,186–26,137)	(701–1,840)	(6,999–27,833)
MSD & MSD-attributable stunting DALYs (1000s)	32,469	531	2,922	74	1,383	409	16,782	19,924	1,083	35,212	2,576	37,789
	(15,784–55,253)	(298–882)	(1,265–5,466)	(41–118)	(677–2,427)	(236–672)	(8,787–27,967)	(11,062–31,430)	(819–1,381)	(18,751–57,532)	(1,882–3,441)	(20,624–60,834)
MSD & MSD-attributable stunting DALYs averted (1000s)	14,714	258	1,522	44	768	216	7,876	9,049	597	16,215	1,307	17,522
	(6,158–26,533)	(129–443)	(607–2,981)	(20–74)	(324–1,439)	(107–374)	(3,479–14,023)	(4,323–15,154)	(342–881)	(7,448–28,294)	(762–1,960)	(8,281–30,166)
MSD & LSD DALYs (1000s)	30,083	605	2,743	89	1,416	470	15,548	18,594	1,264	32,664	2,742	35,406
	(13,024–52,855)	(354–960)	(1,116–5,268)	(50–138)	(695–2,456)	(273–756)	(7,328–26,895)	(9,536–30,059)	(973–1,595)	(16,054–54,830)	(2,047–3,631)	(18,179–58,303)
MSD & LSD DALYs averted (1000s)	13,534	280	1,409	48	751	237	7,236	8,370	654	14,914	1,345	16,259
	(5,388–25,103)	(148–472)	(521–2,865)	(24–81)	(319–1,393)	(122–403)	(3,013–13,337)	(3,882–14,203)	(402–938)	(6,513–26,447)	(812–1,990)	(7,422–28,292)
Total DALYs (1000s)*	39,520	692	3,713	101	1,881	547	20,447	24,585	1,422	43,203	3,250	46,453
	(21,246–62,359)	(409–1,099)	(1,838–6,348)	(58–159)	(960–3,115)	(315–876)	(11,382–31,709)	(14,598–36,643)	(1,095–1,799)	(24,841–66,086)	(2,446–4,259)	(27,293–70,560)
Total DALYs averted (1000s)*	16,864	311	1,789	54	948	265	9,036	10,475	720	18,697	1,534	20,231
	(7,960–28,857)	(168–522)	(796–3,403)	(27–92)	(447–1,720)	(133–447)	(4,497–15,290)	(5,568–16,819)	(447–1,035)	(9,633–31,008)	(949–2,247)	(10,671–32,934)
LSD medical costs (millions US$)	$3,653	$1,207	$814	$215	$1,013	$917	$1,576	$3,548	$2,696	$4,309	$3,511	$7,820
	($1,537–6,445)	($633–2,019)	($350–1,486)	($109–366)	($468–1,862)	($448–1,543)	($786–2,686)	($2,160–5,208)	($1,958–3,697)	($2,459–6,798)	($2,588–4,720)	($5,279–10,942)
LSD medical costs averted (millions US$)	$1,157	$422	$290	$83	$366	$352	$507	$1,174	$988	$1,411	$1,258	$2,669
	($383–2,488)	($170–785)	($91–631)	($32–169)	($134–792)	($133–705)	($192–1,073)	($517–2,163)	($490–1,658)	($605–2,726)	($631–2,147)	($1,236–4,727)
MSD medical costs (millions US$)	$1,436	$549	$329	$95	$405	$425	$582	$1,415	$1,242	$1,644	$1,595	$3,239
	($604–2,533)	($289–920)	($142–601)	($48–161)	($187–744)	($208–715)	($293–984)	($863–2,073)	($897–1,710)	($941–2,583)	($1,180–2,157)	($2,222–4,499)
MSD medical costs averted (millions US$)	$694	$297	$180	$56	$226	$251	$286	$717	$701	$824	$880	$1,704
	($263–1,317)	($142–531)	($70–364)	($25–106)	($89–454)	($106–454)	($130–518)	($378–1,189)	($406–1,058)	($414–1,409)	($513–1,315)	($967–2,666)

	REGION						INCOME CLASS			GAVI		ALL COUNTRIES
	AFRO	AMRO	EMRO	EURO	SEARO	WPRO	LI	LMI	UMI	Eligible	Ineligible	
Other infectious disease deaths from LSD shigella-attributable stunting as a percentage of total shigella deaths	18%	17%	21%	19%	25%	19%	18%	18%	15%	18%	15%	18%
	(4–40%)	(4–36%)	(5–45%)	(5–40%)	(7–49%)	(5–39%)	(5–38%)	(5–38%)	(5–27%)	(5–38%)	(5–29%)	(5–38%)
Other infectious disease deaths from MSD shigella-attributable stunting as a percentage of total shigella deaths	9%	8%	10%	9%	12%	9%	9%	9%	7%	9%	7%	9%
	(3–19%)	(3–17%)	(3–23%)	(3–19%)	(4–24%)	(3–19%)	(3–18%)	(3–17%)	(3–13%)	(3–18%)	(3–13%)	(3–18%)
Other Infectious disease deaths from shigella-attributable stunting as a percentage of total shigella deaths	26%	24%	31%	28%	36%	28%	26%	27%	22%	27%	23%	27%
	(8–54%)	(8–50%)	(11–62%)	(9–55%)	(13–67%)	(10–55%)	(9–52%)	(10–51%)	(10–36%)	(10–52%)	(10–39%)	(10–51%)

* Total deaths and DALYS are the sum of *Shigella* burden attributed to diarrhoea from *Shigella* infection and deaths from other infectious diseases due to *Shigella*-attributable stunting.

NOTE: Though vaccinations occur annually from 2025-2044, impacts are projected over the first five years of the vaccinated child's life. Thus, the last year included in impact estimates is 2049.

Supplementary Table 3. Estimated *Shigella*-attributable mortality and morbidity burden rates without vaccination, burden averted by potential *Shigella* vaccination in children under 5 years of age, and Incremental Cost-Effectiveness Ratios (US$ per Disability-Adjusted Life Year averted by vaccination). Estimates are presented as totals for 102 countries aggregated by WHO region, income classification, Gavi-eligibility and in all 102 countries. Simulation model estimates are presented as mean estimates projected from 2025 (year of introduction) to 2044. All costs and ICERs are presented in 2019 US$. Results from uncertainty analysis are presented as 95% uncertainty intervals in parentheses below model estimates. *LSD: less severe diarrhoea−MSD: moderate−to−severe−diarrhoea−LI: lower income−LMI: lower middle income−UMI: upper middle income−children: children under five years of age−ICER: Incremental Cost-Effectiveness Ratio.*

	REGION						INCOME CLASS			GAVI		ALL COUNTRIES
	AFRO	AMRO	EMRO	EURO	SEARO	WPRO	LI	LMI	UMI	Eligible	Ineligible	
NUMBER OF COUNTRIES	42	17	11	11	9	12	26	46	30	53	49	102
LSD episodes per 100K children	22,592	16,330	12,304	7,684	7,761	6,021	20,494	13,272	9,798	15,663	10,460	13,783
	(9,497–39,842)	(8,545–27,303)	(5,289–22,463)	(3,889–13,043)	(3,585–14,275)	(2,944–10,125)	(9,910–35,441)	(8,070–19,425)	(7,157–13,182)	(8,766–24,713)	(7,706–13,854)	(8,669–20,434)
LSD episodes averted per 100K children	7,213	5,583	4,335	2,925	2,805	2,286	6,590	4,448	3,558	5,153	3,726	4,638
	(2,386–15,518)	(2,247–10,391)	(1,362–9,448)	(1,146–5,957)	(1,028–6,066)	(860–4,578)	(2,421–14,150)	(1,973–8,241)	(1,757–5,941)	(2,162–10,118)	(1,840–6,354)	(2,063–8,557)
MSD episodes per 100K children	7,531	5,443	4,101	2,561	2,587	2,007	6,831	4,424	3,266	5,221	3,487	4,594
	(3,166–13,281)	(2,848–9,101)	(1,763–7,488)	(1,296–4,348)	(1,195–4,758)	(981–3,375)	(3,303–11,814)	(2,690–6,475)	(2,386–4,394)	(2,922–8,238)	(2,569–4,618)	(2,890–6,811)
MSD episodes averted per 100K children	3,668	2,877	2,219	1,499	1,442	1,172	3,354	2,272	1,827	2,628	1,911	2,369
	(1,389–6,971)	(1,380–5,170)	(864–4,472)	(664–2,837)	(572–2,902)	(494–2,118)	(1,448–6,069)	(1,183–3,774)	(1,043–2,732)	(1,286–4,513)	(1,121–2,831)	(1,269–3,905)
LSD-attributable stunting episodes per 100K children	1,157	428	546	136	398	129	1,103	644	252	810	312	630
	(287–2,517)	(130–865)	(145–1,210)	(39–291)	(101–880)	(36–274)	(294–2,267)	(188–1,233)	(83–458)	(240–1,575)	(104–558)	(193–1,196)
LSD shigella-attributable stunting episodes averted per 100K children	370	147	190	51	144	47	357	215	91	267	108	210
	(76–954)	(41–330)	(43–474)	(12–121)	(33–348)	(12–114)	(80–929)	(53–493)	(27–190)	(62–640)	(31–229)	(52–490)
MSD-attributable stunting episodes per 100K children	542	202	257	64	187	60	517	302	118	380	146	296
	(196–1,125)	(77–383)	(86–524)	(24–126)	(69–386)	(22–114)	(204–1,030)	(142–537)	(59–193)	(166–701)	(73–240)	(138–534)
MSD shigella-attributable stunting episodes averted per 100K children	265	108	138	37	104	34	256	155	66	192	78	151
	(85–569)	(38–216)	(43–313)	(13–81)	(34–233)	(12–69)	(86–528)	(61–297)	(29–117)	(71–378)	(34–139)	(59–288)
LSD-attributable stunting deaths per 100K children	5.0	0.2	1.4	<0.1	0.3	<0.1	4.5	2.1	0.1	3.0	0.3	2.0
	(1.2–10.7)	(<0.1–0.5)	(0.4–3.1)	(<0.1–0.2)	(<0.1–0.7)	(<0.1–0.2)	(1.2–9.2)	(0.6–4.1)	(<0.1–0.2)	(0.8–6.1)	(<0.1–0.5)	(0.6–4.1)
LSD-attributable stunting deaths averted per 100K children	1.5	<0.1	0.5	<0.1	0.1	<0.1	1.4	0.6	<0.1	0.9	<0.1	0.6
	(0.3–3.7)	(<0.1–0.2)	(0.1–1.1)	(<0.1–<0.1)	(<0.1–0.3)	(<0.1–<0.1)	(0.3–3.5)	(0.1–1.5)	(<0.1–<0.1)	(0.2–2.2)	(<0.1–0.2)	(0.1–1.5)
MSD-attributable stunting deaths per 100K children	2.4	0.1	0.7	<0.1	0.1	<0.1	2.1	1.0	<0.1	1.4	0.1	1.0
	(0.9–4.9)	(<0.1–0.2)	(0.2–1.4)	(<0.1–<0.1)	(<0.1–0.3)	(<0.1–<0.1)	(0.8–4.2)	(0.4–1.9)	(<0.1–<0.1)	(0.6–2.8)	(<0.1–0.2)	(0.4–1.9)
MSD-attributable stunting deaths averted per 100K children	1.1	<0.1	0.3	<0.1	<0.1	<0.1	1.0	0.4	<0.1	0.7	<0.1	0.4
	(0.3–2.3)	(<0.1–0.1)	(0.1–0.8)	(<0.1–<0.1)	(<0.1–0.2)	(<0.1–<0.1)	(0.3–2.1)	(0.2–0.9)	(<0.1–<0.1)	(0.2–1.3)	(<0.1–0.1)	(0.2–0.9)
Total deaths per 100K children*	29.2	1.5	7.1	0.4	1.3	0.5	25.9	11.6	0.7	17.1	1.7	11.5
	(15.2–46.9)	(0.7–2.6)	(3.3–12.5)	(0.2–0.8)	(0.6–2.3)	(0.2–0.9)	(14.2–40.8)	(6.7–17.6)	(0.5–1.0)	(9.5–26.6)	(1.2–2.4)	(6.5–17.8)
Total deaths averted per 100K children*	12.4	0.6	3.4	0.2	0.7	0.2	11.4	4.9	0.4	7.4	0.8	5.0
	(5.7–21.6)	(0.3–1.2)	(1.4–6.6)	(<0.1–0.5)	(0.3–1.3)	(<0.1–0.4)	(5.5–19.5)	(2.5–8.0)	(0.2–0.6)	(3.7–12.4)	(0.4–1.2)	(2.5–8.3)
MSD only DALYs per 100K children	721	59	176	22	39	20	639	289	32	421	57	290
	(305–1,279)	(33–100)	(68–344)	(12–34)	(18–71)	(11–33)	(295–1,115)	(145–473)	(24–42)	(203–716)	(41–77)	(145–484)
MSD only DALYs averted per 100K children	327	29	92	13	22	11	300	131	18	194	29	134
	(128–610)	(14–50)	(32–190)	(6–22)	(9–41)	(5–19)	(120–556)	(59–225)	(10–26)	(82–347)	(16–43)	(59–236)
	796	63	197	23	44	22	706	320	34	467	60	320

	REGION						INCOME CLASS			GAVI		ALL COUNTRIES
	AFRO	AMRO	EMRO	EURO	SEARO	WPRO	LI	LMI	UMI	Eligible	Ineligible	
MSD & MSD-attributable stunting DALYs per 100K children	(387–1,355)	(35–104)	(85–369)	(13–36)	(21–76)	(12–35)	(370–1,177)	(178–504)	(26–43)	(249–763)	(44–81)	(175–515)
MSD & MSD-attributable stunting DALYs averted per 100K children	361 (151–651)	30 (15–52)	103 (41–201)	13 (6–23)	24 (10–45)	11 (6–20)	331 (146–590)	145 (69–243)	19 (11–28)	215 (99–375)	31 (18–46)	148 (70–255)
MSD & LSD DALYs per 100K children	738 (319–1,296)	71 (42–113)	185 (75–356)	27 (15–42)	45 (22–77)	25 (14–40)	654 (308–1,132)	298 (153–482)	39 (30–50)	433 (213–727)	64 (48–85)	300 (154–494)
MSD & LSD DALYs averted per 100K children	332 (132–616)	33 (17–56)	95 (35–194)	15 (7–25)	24 (10–44)	12 (6–21)	305 (127–561)	134 (62–228)	20 (13–29)	198 (86–351)	32 (19–47)	138 (63–240)
Total DALYs per 100K children*	969 (521–1,530)	82 (48–130)	251 (124–429)	31 (18–49)	59 (30–98)	29 (17–46)	861 (479–1,335)	395 (234–588)	44 (34–56)	573 (329–876)	76 (57–100)	393 (231–598)
Total DALYs averted per 100K children*	414 (195–708)	37 (20–62)	121 (54–230)	16 (8–28)	30 (14–54)	14 (7–23)	380 (189–644)	168 (89–270)	22 (14–32)	248 (128–411)	36 (22–53)	171 (90–279)
LSD medical costs per 100K children	$89,615 ($37,689–158,084)	$142,458 ($74,641–238,250)	$54,985 ($23,647–100,371)	$66,023 ($33,374–112,127)	$31,922 ($14,750–58,679)	$48,170 ($23,532–81,075)	$66,348 ($33,073–113,058)	$56,935 ($34,656–83,576)	$84,226 ($61,174–115,497)	$57,127 ($32,609–90,130)	$82,317 ($60,675–110,659)	$66,227 ($44,708–92,668)
LSD medical costs averted per 100K children	$28,374 ($9,392–61,031)	$49,738 ($20,043–92,655)	$19,560 ($6,152–42,601)	$25,347 ($9,927–51,682)	$11,542 ($4,233–24,949)	$18,487 ($6,969–37,057)	$21,326 ($8,080–45,150)	$18,843 ($8,299–34,709)	$30,862 ($15,312–51,799)	$18,701 ($8,023–36,147)	$29,496 ($14,792–50,339)	$22,601 ($10,466–40,034)
MSD medical costs averted per 100K children	$35,223 ($14,814–62,138)	$64,744 ($34,039–108,577)	$22,249 ($9,568–40,613)	$29,169 ($14,669–49,513)	$12,751 ($5,892–23,438)	$22,320 ($10,903–37,567)	$24,482 ($12,317–41,413)	$22,713 ($13,848–33,264)	$38,794 ($28,012–53,431)	$21,795 ($12,471–34,246)	$37,389 ($27,670–50,559)	$27,428 ($18,814–38,102)
MSD medical costs averted per 100K children	$17,021 ($6,452–32,302)	$34,987 ($16,767–62,650)	$12,187 ($4,744–24,598)	$17,242 ($7,619–32,450)	$7,109 ($2,816–14,294)	$13,206 ($5,566–23,879)	$12,036 ($5,467–21,814)	$11,502 ($6,058–19,083)	$21,911 ($12,675–33,045)	$10,921 ($5,486–18,683)	$20,637 ($12,016–30,827)	$14,431 ($8,193–22,576)
ICER shigella MSD ($/DALY)	$313 ($126–732)	$7,310 ($2,822–14,915)	$1,948 ($716–4,670)	$21,653 ($9,460–42,945)	$7,619 ($3,058–16,295)	$31,850 ($14,557–63,466)	$270 ($107–625)	$1,017 ($509–2,077)	$17,396 ($10,083–32,163)	$518 ($242–1,104)	$10,196 ($5,897–18,238)	$1,317 ($634–2,726)
ICER shigella MSD & MSD stunting ($/DALY)	$276 ($115–612)	$6,913 ($2,669–13,910)	$1,687 ($643–3,686)	$20,507 ($9,019–41,245)	$6,701 ($2,781–13,904)	$30,111 ($14,021–60,305)	$238 ($100–521)	$903 ($462–1,758)	$16,575 ($9,688–30,362)	$458 ($221–926)	$9,556 ($5,622–16,904)	$1,170 ($595–2,359)
ICER shigella MSD & LSD ($/DALY)	$206 ($25–541)	$4,726 ($953–10,564)	$1,614 ($538–3,872)	$16,511 ($6,471–33,800)	$6,292 ($2,499–13,401)	$25,701 ($11,356–51,275)	$183 ($42–457)	$828 ($388–1,667)	$13,455 ($7,699–24,381)	$398 ($159–863)	$8,243 ($4,639–14,194)	$1,089 ($512–2,185)
ICER shigella MSD & LSD & MSD & LSD stunting ($/DALY)	$161 ($18–383)	$4,237 ($847–9,307)	$1,210 ($434–2,575)	$14,840 ($5,773–30,191)	$4,893 ($2,042–9,942)	$22,990 ($10,200–46,562)	$143 ($33–332)	$644 ($304–1,169)	$12,193 ($6,872–21,231)	$308 ($124–589)	$7,191 ($4,142–12,250)	$849 ($423–1,575)

* Total deaths and DALYS are the sum of *Shigella* burden attributed to diarrhoea from *Shigella* infection and deaths from other infectious diseases due to *Shigella*-attributable stunting.

NOTE: Though vaccinations occur annually from 2025-2044, impacts are projected over the first five years of the vaccinated child's life. Thus, the last year included in impact estimates is 2049.

Supplementary Table 4. Estimated Incremental Cost-Effectiveness Ratios (Net costs per Disability-Adjusted Life Year averted) for potential *Shigella* vaccination in children under 5 years of age. Incremental Cost-Effectiveness Ratios were calculated from estimates for 102 countries aggregated by WHO region, income classification, Gavi-eligibility and in all 102 countries. Simulation model estimates are presented as median estimates projected from 2025 (year of introduction) to 2044. All ICERs are presented in 2019 US$. Results from uncertainty analysis are presented as Inter Quartile Ranges (IQR) in parentheses below model estimates. *LSD: less severe diarrhoea−MSD: moderate−to−severe−diarrhoea−LI: lower income−LMI: lower middle income−UMI: upper middle income−children: children under five years of age−ICER: Incremental Cost-Effectiveness Ratio.*

	REGION						INCOME CLASS			GAVI		ALL COUNTRIES
	AFRO	AMRO	EMRO	EURO	SEARO	WPRO	LI	LMI	UMI	Eligible	Ineligible	
NUMBER OF COUNTRIES	42	17	11	11	9	12	26	46	30	53	49	102
1) ICER *Shigella* MSD episodes ($/DALY)	$272	$6,757	$1,692	$19,815	$6,816	$29,328	$236	$918	$16,382	$462	$9,624	$1,177
	($207–373)	($4,929–8,989)	($1,226–2,394)	($15,017–26,221)	($5,175–9,184)	($22,439–38,028)	($180–316)	($735–1,196)	($13,701–19,613)	($365–607)	($8,100–11,472)	($948–1,547)
2) ICER Shigella MSD & LSD episodes ($/DALY)	$180	$4,386	$1,428	$15,279	$5,670	$23,597	$160	$758	$12,858	$355	$7,828	$981
	($127–258)	($2,893–6,273)	($996–1,989)	($11,351–20,221)	($4,324–7,573)	($18,302–30,986)	($115–227)	($601–983)	($10,657–15,299)	($275–479)	($6,539–9,387)	($787–1,291)
3) ICER Shigella MSD & LSD episodes & MSD & LSD stunting cases ($/DALY)	$145	$3,925	$1,106	$13,857	$4,497	$21,187	$129	$604	$11,641	$285	$6,894	$790
	($99–206)	($2,614–5,646)	($797–1,505)	($10,340–18,244)	($3,420–5,918)	($16,411–27,468)	($92–177)	($484–752)	($9,710–13,966)	($219–369)	($5,761–8,185)	($635–1,005)
MSD+S only: ICERs Shigella MSD episodes & MSD stunting cases ($/DALY)	$242	$6,423	$1,495	$18,735	$5,974	$27,652	$213	$822	$15,640	$413	$9,062	$1,056
	($191–332)	($4,671–8,567)	($1,115–2,079)	($14,260–24,776)	($4,651–8,087)	($21,299–36,045)	($165–281)	($673–1,059)	($13,065–18,680)	($332–539)	($7,624–10,698)	($868–1,362)

NOTE: Though vaccinations occur annually from 2025-2044, impacts are projected over the first five years of the vaccinated child's life. Thus, the last year included in impact estimates is 2049

Supplementary Figure 1. Conceptual diagram of burden model. Red-outlined compartments represent the model components added in this model iteration when comparing to the previous model iteration[13]. *MSD: moderate-to-severe diarrhoea; LSD: less severe diarrhoea; YLD: Years Lived with Disability; YLL: Years of Life Lost; WHO: World Health Organization[9]; DHS: Demographic and Health Surveys[14]; GBD: Global Burden of Disease[15]; GEMS 1A: Global Enteric Multicenter Study.*[6]

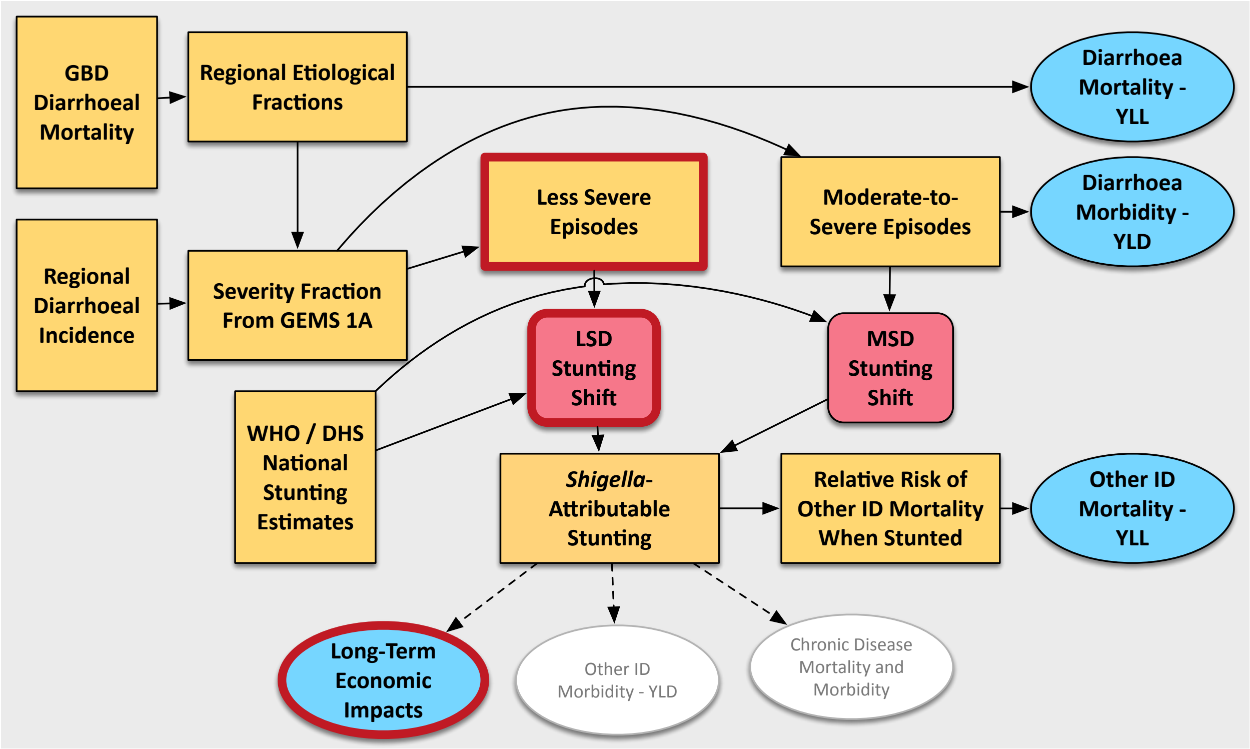

Supplement: Supplementary appendix [file mmc1.pdf]
